# Antioxidant and Anti-Inflammatory Activities of Unexplored Brazilian Native Fruits

**DOI:** 10.1371/journal.pone.0152974

**Published:** 2016-04-06

**Authors:** Juliana Infante, Pedro Luiz Rosalen, Josy Goldoni Lazarini, Marcelo Franchin, Severino Matias de Alencar

**Affiliations:** 1 Department of Agri-food Industry, Food and Nutrition, ‘Luiz de Queiroz’ College of Agriculture, University of São Paulo, Pádua Dias Avenue, P.O. Box. 9, 13418-900, Piracicaba, SP, Brazil; 2 Piracicaba Dental School, Department of Physiological Sciences, University of Campinas, 901, Limeira Avenue, 13414-903, Piracicaba, SP, Brazil; Islamic Azad University-Mashhad Branch, Mashhad, Iran, ISLAMIC REPUBLIC OF IRAN

## Abstract

Brazilian native fruits are unmatched in their variety, but a poorly explored resource for the development of food and pharmaceutical products. The aim of this study was to evaluate the phenolic composition as well as the antioxidant and anti-inflammatory activities of the extracts of leaves, seeds, and pulp of four Brazilian native fruits (*Eugenia leitonii*, *Eugenia involucrata*, *Eugenia brasiliensis*, and *Eugenia myrcianthes*). GC—MS analyses of the ethanolic extracts showed the presence of epicatechin and gallic acid as the major compounds in these fruits. Antioxidant activity was measured using synthetic DPPH free-radical scavenging, β-carotene bleaching assay, and reactive oxygen species (ROO·, O_2_·^−^, and HOCl). The fruit extracts also exhibited antioxidant effect against biologically relevant radicals such as peroxyl, superoxide, and hypochlorous acid. In general, the pulps were the fruit fractions that exhibited the lowest antioxidant activities, whereas the leaves showed the highest ones. The anti-inflammatory activity was assessed in an *in vivo* model using the carrageenan-induced neutrophil migration assay, which evaluates the inflammatory response in the acute phase. The pulp, seeds, and leaves of these fruits reduced the neutrophil influx by 40% to 64%. Based on these results, we suggest that the anti-inflammatory activity of these native fruits is related to the modulation of neutrophil migration, through the inhibition of cytokines, chemokines, and adhesion molecules, as well as to the antioxidant action of their ethanolic extracts in scavenging the free-radicals released by neutrophils. Therefore, these native fruits can be useful to produce food additives and functional foods.

## Introduction

The considerable biodiversity of the Brazilian ecosystem represents a valuable and unexploited source of foods, extracts, and compounds with the potential to improve human health and wellness [1]. Brazil has the most diverse flora in the world, which offers unprecedented opportunities in the discovery of edible plant tissues rich in nutrients and bioactive compounds that can prevent and treat chronic non-communicable diseases (NCD), such as metabolic syndrome and cancer. NCD affect as many as 3 billion people worldwide, and constitute the major pathogenic conditions in the aging population. Unique fruits native to Brazil certainly represent a promising source for the discovery of bioactive extracts and compounds.

The chemical profiles (e.g. polyphenols, carotenoids, and fiber) and health-promoting properties (e.g. antioxidant, anti-inflammatory, anti-obesogenic, antitumorigenic, and probiotic activities) of many native plants remain unknown and they are rarely included in modern diets, especially in urban areas [2–4].

Numerous epidemiological analyses, cell and animal studies, as well as human intervention trials consistently support the argument that diets rich in fruits and vegetables can reduce the risk and severity of many NCD, such as atherosclerosis, cancer, diabetes, and neurodegenerative disorders [5,6]. Moreover, projections that food production/availability must double by 2050 in order to feed the estimated global population of 9–10 billion individuals also have resulted in greater attention to the need for diversifying food systems and developing sustainable practices that will enhance productivity and efficiency of post-harvest processing, and consequently decrease food waste [7].

The goal of this study was to determine the antioxidant and anti-inflammatory activities and the presence of phenolic compounds in leaves, seeds, and pulps of four Brazilian native unexplored fruits, which are rich sources of bioactive compounds.

## Materials and Methods

### Chemicals

The following chemicals were used in this study: Folin-Ciocalteu reagent (Dinâmica Química Contemporânea, Diadema, SP, Brazil); sodium carbonate and ethanol (Synth, Diadema, SP, Brazil); the standards (±)-6-hydroxy-2,5,7,8-tetramethylchroman-2-carboxylic acid (Trolox), sinapic acid, gallic acid, caffeic acid, *p*-coumaric acid, *m*-coumaric, cinnamic acid, ferulic acid, myricetin, mangiferin, procyanidin B_1_, procyanidin B_2_, (–)-catechin, (–)-epicatechin, epicatechin-3-*O*-gallate, myrtillin, kuromanin, peonidin, oenin, petunidin, and quercetin; and the reagents 1,1-diphenyl-2-picrylhydrazyl (DPPH·), monobasic and dibasic potassium phosphate, β-carotene, linoleic acid, Tween 40, fluorescein sodium salt, 2,2′-azobis(2-methylpropionamidine) dihydrochloride (AAPH), β-nicotinamide adenine dinucleotide (NADH), phenazine methosulfate (PMS), nitrotetrazolium blue chloride (NBT), sodium hypochlorite solution (NaOCl), rhodamine 123, N-methyl-N-(trimethylsilyl)trifluoroacetamide (MSTFA), carrageenan, and dexamethasone were purchased from Sigma-Aldrich (St. Louis, MO, USA). The Panoptic Staining Kit was obtained from Laborclin ^®^.

### Ethics statements

Prior to the conduction of this work, we obtained the authorization of the Conselho de Gestão do Patrimônio Genético (CGEN) to access the genetic heritage of the Brazilian native fruit species here studied (CGEN Process no. 010907/2014-9).

The animal study was carried out in strict accordance with the recommendations in the Guidelines for the Care and Use of Laboratory Animals. The protocol was approved by the Ethics Committee on Animal Research (CEUA) of the University of Campinas (UNICAMP), protocol no. 3325–1.

### Plant materials

Fruits and leaves belonging to the kingdom Plantae, class Equisetopsida, subclass Magnoliidae, superorder Rosanae, order Myrtales, family Myrtaceae, genus Eugenia, species *Eugenia brasiliensis* Lam., *Eugenia involucrata* DC, *Eugenia myrcianthes* Nied, and *Eugenia leitonii* D. Legrand were collected, from October 2011 to March 2012, in two orchards located in the state of São Paulo, Brazil (22°16′13.3″S, 47°32′08.2″W and -23°27′53.94″S, -45°42′31.88″W). Whole leaves and ripe fruits were selected, all free from injuries, transported under refrigeration, and washed under running tap water. The fruits were separated into seeds and pulp and all the material so obtained was frozen, lyophilized, and stored at -18°C. Vouchers of the aforementioned species were deposited at the herbarium of the “Luiz de Queiroz” College of Agriculture, University of São Paulo, Piracicaba, SP, as follows: *E*. *brasiliensis* (ESA056895), *E*. *involucrata* (ESA000631), *E*. *myrcianthes* (ESA002665), and *E*. *leitonii* (ESA123645).

### Preparation of the ethanolic extracts of native fruit species

The extracts of leaves, seeds, and pulp of the four native fruit species were prepared in triplicate. For each extract, 2 g of the lyophilized material was ground to a fine powder, extracted with 20 mL 80% ethanol (v/v), sonicated (180 W, 30 min, at room temperature), and centrifuged (5000 x g for 15 min). The supernatant was filtered and employed in the analyses of antioxidant and anti-inflammatory activities, as well as phenolic composition.

### Total phenolic content

Total phenolic content was analyzed according to the method of Singleton et al. [8], using 2.5 mL of the Folin-Ciocalteau reagent (10%), 0.5 mL of the extracts diluted, and 2.0 mL of 4% sodium carbonate. After 2 h, the absorbance of the mixtures was measured at 740 nm using a spectrophotometer UV-mini 1240 (Shimadzu Corp., Kyoto, Japan), and the results are expressed in mg of gallic acid per g of lyophilized material.

### Chromatographic analysis

#### Removal of outliers from samples using solid phase extraction (SPE)

The solid phase extraction (SPE) technique was employed for the removal of sugars, which could mask the compounds of interest. This technique has been increasingly used because it is quick, efficient, and requires very small volumes of samples and solvents. LC-18 SPE cartridges (2 g, Supelco, Bellefonte, PA, USA) were conditioned with methanol and acidic water (pH = 2.0). Subsequently, 4 mL of each native fruit species ethanolic extract once again evaporated and redissolved in 4 mL of water were added to their respective cartridges. After the extract completely passed through, the column was washed with sufficient acidic water to remove the sugars. Compounds of interest were eluted with methanol into coded glass vials and their bands were identified under UV light at 366 nm (Cole-Parmer^®^).

#### Derivatization—formation of trimethylsilyl derivatives (TMS)

Prior to the gas chromatography-mass spectrometry (GC-MS) analysis, the samples were submitted to a crucial stage called derivatization. GC-MS is only useful for the analysis of gases, as well as volatile and thermally stable substances. Samples not showing this profile, presenting high molecular weight compounds, and/or strongly polar functional groups, require a derivatization procedure, a reaction which transforms a substance of interest into a product of similar chemical structure, called a derivative, which presents characteristics suitable for analysis. Chemical derivatization is widely used to reduce the polarity of functional groups and facilitate their separation during GC-MS analysis.

The fractions obtained after purification were added to 100 μL of derivatizing reagent MSTFA. The reaction mixture was homogenized and incubated at 70°C for 10 min. The reagent was evaporated under a stream of nitrogen and trimethylsilyl (TMS) derivatives were rediluted in hexane (800 μL). After homogenization, the supernatant was transferred to a vial and injected into the GC-MS system.

#### Gas chromatography-mass spectrometry (GC-MS)

GC-MS analyses of the ethanolic extracts of the native fruit species were performed on a gas chromatograph GC 2010 (Shimadzu Corp., Kyoto, Japan) coupled to a mass spectrometer QP 2010 Plus (Shimadzu Corp., Kyoto, Japan). Derivatized samples were separated using a capillary column (RTX-5MS 30 m × 0.25 mm × 0.25 μm). The temperature program started at 80°C (1 min), increasing at 20°C/min to 250°C, remaining at 250°C for 1 min (9 min 30 s), increasing at 6°C/min to 300°C, remaining at 300°C for 5 min (13 min 20 s), increasing at 15°C/min to 310°C, remaining at 310°C for 5 min (5 min 40 s), increasing at 20°C/min to the final temperature of 320°C, remaining at 320°C for 10 min (10 min 30 s), totaling 40 min of analysis. Helium was used as the carrier gas, the injector temperature was 280°C, and the injection volume was 0.3 μL in splitless mode. The interface was maintained at 280°C and the detector was operated in the scanning mode (m/z 40–800). Data integration was performed using the LabSolutions-GCMS software. Flavonoids, phenolic acids, and derivatives were identified by comparing their retention time and ion fragmentation with coded and authentic standards (sinapic acid, gallic acid, caffeic acid, *p*-coumaric acid, *m*-coumaric acid, cinnamic acid, ferulic acid, myricetin, mangiferin, procyanidin B_1_, procyanidin B_2_, (–)-catechin, (–)-epicatechin, epicatechin-3-*O*-gallate, myrtillin, kuromanin, peonidin, oenin, petunidin, and quercetin), eluted under the same conditions and compared with data from the Wiley 138 library [9].

### Antioxidant activity assays

#### DPPH free-radical scavenging

The scavenging activity of the ethanolic extracts of the native fruit species was carried out according to Tiveron et al. [10]. The mixture was composed of 0.5 mL of sample and 3.3 mL of 0.05 mM DPPH ethanolic solution, monitored at 517 nm using a spectrophotometer UV-mini 1240 (Shimadzu Corp., Kyoto, Japan) at intervals of 20 min up to constant values of absorbance. The results are expressed as EC_50_ (minimum concentration of plant material required to reduce the initial concentration of DPPH by 50%).

#### β-carotene bleaching

β-carotene bleaching was employed following Emmons et al. [11] with modifications. In a test tube, an aliquot of 3.0 mL of an emulsion consisting of β-carotene, linoleic acid, Tween, and aerated distilled water was mixed with 50 μL of the diluted ethanolic extracts of the native fruit species and incubated in water bath at 50°C. After 120 min, the absorbance was read at 470 nm using a spectrophotometer UV-mini 1240 (Shimadzu Corp., Kyoto, Japan). The antioxidant activity of the samples is expressed as Trolox equivalent (TE) (μmol TE/g of lyophilized material).

#### Peroxyl radical scavenging capacity (PSC)

Peroxyl radical (ROO·) scavenging capacity was measured according to Melo et al. [4]. This assay monitored the antioxidant action of the ethanolic extracts of the native fruit species on the fluorescence decay by ROO·-induced oxidation of fluorescein and is expressed as the oxygen radical absorbance capacity (ORAC). Aliquots of 30 μL of standard, control, or samples, 60 μL of 508.25 nM fluorescein, and 110 μL of a solution of 76 mM AAPH were mixed in a microplate. The solutions were diluted with potassium phosphate buffer 75 mM (pH 7.4), also used as a blank. The reaction was performed at 37°C, and the readings were made each minute for 2 h, at excitation and emission wavelengths of 485 and 528 nm, respectively, using a microplate reader SpectraMax^®^ M3. Trolox was used as a standard at concentrations ranging from 12.5 to 400 μM, and the results are expressed as Trolox equivalent (TE) (μmol TE/g of lyophilized material).

#### Superoxide anion radical scavenging capacity

The capacity of the ethanolic extracts of the native fruit species to scavenge the superoxide radical (O_2_·^−^), generated by the NADH/PMS system, was measured according to Melo et al. [4], with modifications. In a microplate, we added 166 μM NADH, 107.5 μM NBT, different concentrations of the extracts, and 2.7 μM PMS, dissolved in 19 mM potassium phosphate buffer (pH 7.4) to a final volume of 300 μL. The assay was conducted at 25°C, and after 5 min, the absorbance was measured at 560 nm using a microplate reader SpectraMax^®^ M3. A control was prepared replacing the sample with buffer, and a blank corresponding to each sample dilution was prepared by replacing PMS and NADH with buffer. The results are expressed as EC_50_ [minimum quantity (mg/mL) of the sample required to quench 50% of the superoxide radical].

#### Hypochlorous acid scavenging capacity

The hypochlorous acid (HOCl) scavenging capacity was determined based on the effect of the ethanolic extracts of the native fruit species on HOCl-induced oxidation of dihydrorhodamine 123 (DHR) to rhodamine 123, according to Melo et al. [4], with modifications. The HOCl was prepared using a solution of 1% NaOCl, with pH adjusted to 6.2 with the addition of 10% H_2_SO_4_ solution. The concentration of this solution diluted in 100 mM phosphate buffer (pH 7.4) was measured spectrophotometrically at 235 nm using the molar absorption coefficient 100 M^-1^ cm^-1^. A stock solution of DHR diluted in 1.15 mM dimethylformamide was used to prepare the working solutions in phosphate buffer immediately before the analysis. The reaction mixture contained the extracts at different concentrations, 100 mM phosphate buffer (pH 7.4), 1.25 μM DHR, and 5 μM HOCl to a final volume of 300 μL. The assay was conducted at 37°C in a microplate reader (SpectraMax^®^ M3), and the fluorescence signal was obtained immediately at the emission wavelength of 528 ± 20 nm with excitation at 485 ± 20 nm. The results are expressed as EC_50_ (mg/mL) of the sample.

### Evaluation of anti-inflammatory activity

#### Animals

Male Balb/c albino mice (20–25 g), specific-pathogen free (SPF), were purchased from CEMIB/UNICAMP (Multidisciplinary Center for Biological Research, SP, Brazil) and used as experimental animals. The mice were maintained in a room with controlled temperature (22–25°C), in a 12 h light/12 h dark cycle, 40–60% humidity, with food (standard pellet diet) and water *ad libitum*. The animals fasted for 8 h before the administration of the ethanolic extracts of the native fruit species.

#### Neutrophils migration in the peritoneal cavity *in vivo*

In order to determine the neutrophil migration to the peritoneal cavity, the ethanolic extracts of the native fruit species (500 mg/kg), dexamethasone (2 mg/kg), or vehicle were administered orally (v.o.) by intra-gastric gavage to the mice 60 min before the administration of the inflammatory stimuli by intraperitoneal injection of carrageenan at 500 μg/cavity. The vehicle (0.9% NaCl) was used as the negative control. Mice were euthanized 4 h after the challenge (carrageenan) administration. The peritoneal cavity cells were harvested by washing the cavity with 3 mL of phosphate buffered saline (PBS) containing EDTA. The volumes recovered were approximately 95% of the injected volume, and similar in all experimental groups. In order to count the total number of cells, a Newbauer chamber was used. Smears were prepared using a cytocentrifuge Cytospin 3 (Shandon Lipshaw, Pittsburgh, PA, USA) and stained using the Panoptic Staining Kit. The different cells were counted (up to 100 cells) using an optical microscope (1000x). The results are presented as the number of neutrophils per cavity [4].

### Statistical analyses

Data are expressed as mean ± standard deviation of the mean (M±SD) and statistical comparison between groups was carried out utilizing analysis of variance (ANOVA) followed by Tukey’s test. Significance was accepted when *p* ≤ 0.05.

## Results and Discussion

### Analysis of total phenolic compounds

The content of total phenolic compounds in the ethanolic extracts of leaves, seeds, and pulp of the four studied species of native fruits of the genus *Eugenia* sp. is shown in Table 1. Except for the seeds of *E*. *leitonii* (120.67 mg AG/g), the leaves were the parts that exhibited the highest reducing power.

**Table 1:** Content of total phenolic compounds in the ethanolic extracts of leaves, seeds, and pulp of four Brazilian native fruit species.

Species	Equivalent mg gallic acid/g*		
	Leaves	Seeds	Pulp
*E*. *leitonii*	88.62 ± 2.43^bB^	120.67 ± 4.38^aA^	15.18 ± 0.79^dC^
*E*. *involucrata*	70.19 ± 1.88^aC^	22.75 ± 0.50^bC^	18.36 ± 0.66^cB^
*E*. *brasiliensis*	73.25 ± 3.41^aC^	42.60 ± 1.90^bB^	26.69 ± 1.22^cA^
*E*. *myrcianthes*	100.29 ± 3.29^aA^	11.34 ± 0.49^cD^	17.80 ± 0.89^bB^

* Means of triplicates.Different lower case letters in the same line and upper case letters in the same column differ statistically by the Tukey’s test (*p* < 0.05).

In general, the seeds had higher contents of phenolic compounds than the pulps. In spite of this, the reducing power of the pulps assessed was the same or superior to cultivars of blackberry, raspberry, and strawberry, which showed contents ranging from 9.46 to 15.30 mg AG/g [12]. We observed, with few exceptions, the following trend regarding the reducing power in the present study, analyzed using the Folin-Ciocalteau method: leaves > seeds > pulp (Table 1). It is important to point that, in addition to phenolics, compounds belonging to other classes, such as carotenoids, can also contribute to antioxidant activity and redox potential of plant extracts [13].

### Gas chromatography mass spectrometry

**Fig 1:**
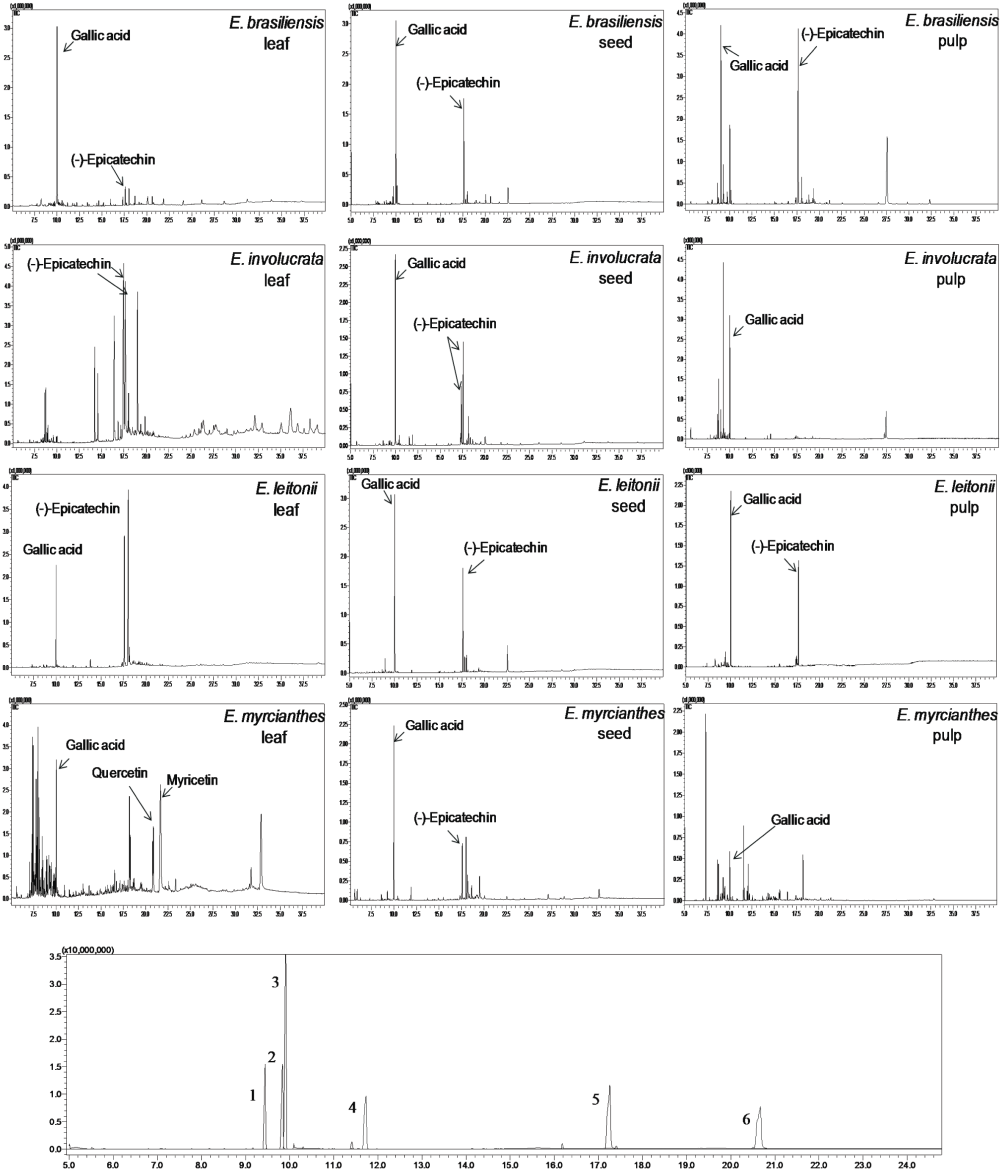
GC/MS chromatograms of extracts of leaves, seeds, and pulp of four Brazilian native fruit species and standards of phenolic compounds. (1) *m*-coumaric acid, (2) *p*-coumaric acid, (3) gallic acid, (4) sinapic acid, (5) (-)-epicatechin, (6) quercetin.

The two main phenolic compounds identified by GC-MS were gallic acid and (–)-epicatechin, which were present in almost all samples of leaves, seeds, and pulp analyzed. Except for the pulp and leaves of *E*. *myrcianthes*, these two compounds reached almost 70% of the total phenolic compounds (Fig 1 and Table 2).

**Table 2:** Phenolic composition of the ethanolic extracts of leaves, seeds, and pulp of four Brazilian native fruit species determined by GC-MS.

Phenolic compound	Area of the component (%)*											
	*Eugenia brasiliensis*			*Eugenia involucrata*			*Eugenia myrcianthes*			*Eugenia leitonii*		
	Leaves	Seeds	Pulp	Leaves	Seeds	Pulp	Leaves	Seeds	Pulp	Leaves	Seeds	Pulp
Cinnamic acid	–	–	–	–	–	–	–	–	0.43	–	–	–
Protocatechuic acid	–	–	–	–	–	–	–	–	–	–	–	–
*p*-coumaric acid	–	–	–	0.26	–	–	–	–	–	–	–	–
*m*-coumaric acid	–	–	0.06	–	–	0.83	–	–	–	–	–	–
Gallic acid	69.4	45.56	9.02	–	42.42	24.55	4.67	33.89	6.68	13.36	12.04	51.31
Sinapic acid	–	–	–	–	–	–	0.03	–	–	–	–	–
6,7-dihydroxycoumarin β-D-glucopyranoside	–	–	–	–	–	–	–	–	1.67	–	–	–
(–)-epicatechin	7.1	26.58	29.38	49.43	33.52	-	0.76	14.23	–	31.25	63.36	39.01
Kaempferol	–	–	–	–	–	–	0.2	–	–	–	–	–
Quercetin	–	–	0.12	–	–	–	4.05	–	–	–	–	–
Myricetin	–	–	–	–	–	–	15.31	–	–	–	–	–

*–non-detected

Antimicrobial, antiviral, and antioxidant activities have been atributed to gallic acid. This compound can also be promising in the treatment of cancer, mainly due to its selective cytoxicity when easily inducing apoptosis in tumor cells [14].

Flavonols, such as catechin and epicatechin, have different mechanisms of action depending on the concentration they reach in the target tissue. On the one hand, micromolar levels would suffice for scavenging free-radicals and metals that induce oxidative stress. On the other hand, in nanomolar levels, more complex and specific chemical interactions, such as the inhibition of free radical generating-enzymes (NADPH oxidase) and regulation of cell signaling, would better explain their beneficial function in the human body. In intestine cells, (–)-epicatechin, along with other phenolic compounds, can also reduce the transient increase in oxidants associated with the activation of tumor necrosis factor (TNFα) signaling [15].

### Antioxidant activity

To the best of our knowledge, this is the first time that antioxidant activity of the leaves, seeds, and pulps of these four Brazilian native fruit species is assessed using different mechanisms of action. The antioxidant activities determined by DPPH free-radical scavenging, β-carotene bleaching, ORAC, superoxide anion radical scavenging, and hypochlorous acid methods are shown in Table 3.

**Table 3:** Antioxidant activity of the ethanolic extracts of leaves, seeds, and pulp of four Brazilian native fruit species measured using synthetic DPPH free-radical scavenging, β-carotene bleaching, and reactive oxygen species (ROO·, O_2_·^−^, and HOCl) assays.

Sample	DPPH (EC_50,_ μg/mL)	β -carotene bleaching (μmol Trolox/g)	ORAC (μmol TE/g)	Superoxide anion (EC_50,_ mg/mL)	Hypochlorous acid (EC_50,_ μg/mL)
*Eugenia brasiliensis*					
Leaves	140.33 ± 3.7^i^	5.42 ± 0.4^b^	757.32 ± 34.7^b^	0.40 ± 0.00^e^	45.81 ± 0.53^g^
Seeds	196.55 ± 3.9^h^	3.89 ± 0.1^cd^	211.84 ± 9.3^g^	0.59 ± 0.02^e^	136.10 ± 8.82^c^
Pulp	472.37 ± 3.2^e^	4.24 ± 0.1^cd^	477.45 ± 23,3^ed^	2.15 ± 0.15^c^	42.67 ± 2.10^g^
*Eugenia involucrata*					
Leaves	191.50 ± 0.3^h^	7.18 ± 0.6^a^	1,393.3 ± 69.6^a^	–	13.84 ± 0.66^j^
Seeds	346.70 ± 14.1^f^	3.38 ± 0.3^de^	168.66 ± 4.9^gh^	1.11 ± 0.04^d^	152.57 ± 4.45^b^
Pulp	988.52 ± 22.4^a^	4.56 ± 0.1^bc^	321.74 ± 9.8^f^	–	58.31 ± 4.34^f^
*Eugenia myrcianthes*					
Leaves	83.80 ± 3.0^jk^	5.27 ± 0.3^b^	644.31 ± 25.4^c^	0.37 ± 0.02^e^	39.95 ± 0.83^gh^
Seeds	734.51 ± 21.2^c^	1.13 ± 0.0^f^	96.24 ± 4.7^h^	6.28 ± 0.33^a^	209.49 ± 6.84^a^
Pulp	597.51 ± 9.5^d^	1.59 ± 0.1^f^	127.62 ± 5.8^h^	4.02 ± 0.33^b^	136.25 ± 5.58^c^
*Eugenia leitonii*					
Leaves	112.38 ± 1.8^ji^	3.01 ± 0.5^e^	417.71 ± 16.7^e^	0.48 ± 0.00^e^	29.55 ± 1.86^hi^
Seeds	69.55 ± 1.7^k^	7.07 ± 0.5^a^	514.76 ± 21.5^d^	0.26 ± 0.01^e^	18.14 ± 0.70^ij^
Pulp	792.43 ± 8.4^b^	0.74 ± 0.0^f^	102.82 ± 7.8^h^	2.67 ± 0.33^c^	109.92 ± 1.63^d^

* Letters compare values in the same column by the Tukey’s test (*p* < 0.05).

Regarding the scavenging activity of the radical DPPH, the best results were registered for the ethanolic extracts of *E*. *leitonii* seeds (EC_50_ = 69.55 μg/mL) and *E*. *myrcianthes* leaves (EC_50_ = 83.80 μg/mL), whereas for Trolox, a water-soluble vitamin E analog, EC_50_ was 21.30 μg/mL. In general, the pulps were the plant parts that exhibited the lowest activities. The Asian loquat fruit (*Eriobotrya japonica* Lindl.) showed the same pattern, i.e. the antioxidant activitiy of skin extracts was higher than that of the pulp extracts [16].

The ethanolic extracts of *E*. *leitonii* seeds and *E*. *involucrata* leaves presented the highest antioxidant activities evaluated using radicals generated in a β-carotene-linoleic acid system, 7.07 and 7.18 μmol Trolox/g, respectively. Similarly to the results obtained using the DPPH free-radical scavenging assay, the ethanolic extracts of pulps of all the fruits analyzed by the β-carotene method exhibited lower activities compared with the ethanolic extracts of leaves of the respective species.

The highest activity using the ORAC method, which measures the capacity of deactivation of peroxyl radical, was found for the ethanolic extracts of *E*. *involucrata* leaves (1,393.3 μmol TE/g), whereas the pulps exhibited ORAC values ranging from 102.82 to 477.45 μmol TE/g. In a study assessing 40 varieties of blueberry, ORAC values ranged from 196 to 528 μmol TE/g [17]. For other traditionally known fruits such as guava, grape, cherry, and strawberry, ORAC values ranged from 64.28 to 401.91 μmol TE/g [18,19], which corroborates the great antioxidant potential of the native fruits hereby studied.

As to the antioxidant activities determined by superoxide anion radical scavenging, the lowest values of EC_50_ were obtained for the ethanolic extracts of *E*. *leitonii* seeds and leaves (0.26 and 0.48 mg/mL, respectively), *E*. *myrcianthes* leaves (0.37 mg/mL), and *E*. *brasiliensis* leaves and seeds (0.40 and 0.59 mg/mL, respectively). Based on these results, *E*. *leitonii* seeds display antioxidant activity similar to that of epicatechin, which has an EC_50_ of 0.23 mg/mL [4]. Once more, the pulps showed the lowest activities for deactivating this reactive oxygen species (ROS).

With regard to the capacity of deactivation of hypochlorous acid, the ethanolic extracts of *E*. *Involucrata* leaves and *E*. *leitonii* seeds were the samples which exhibited the highest scavenging ability (EC_50_ = 13.84 μg/mL and 18.14 μg/mL, respectively). *Eugenia brasiliensis* displayed a unique behavior, since it was the only studied species in which the pulp exhibited antioxidant activity statistically equal to that of the leaves (EC_50_ = 42.67 and 45.81 μg/mL, respectively). However, in general, the leaves showed high capacity of deactivation of hypochlorous acid. The leaves have high antioxidant activity because many ROS, potentially harmful to cells, are formed during the process of photosynthesis [20].

Reactive species such as peroxyl, superoxide anion, and hypochlorous acid have highly unstable structure and display electrophilic character. Therefore, they are capable of rapidly attacking molecules presenting high electron density, such as the insaturated fatty acids found in foods and human body, as well as biomolecules (proteins, DNA, and RNA), and consequently impair vital functions in the human body. Thus, juices or foods prepared with a mix of the pulp and leaves of the four Brazilian native fruit species herein studied can be an interesting option to strengthen the antioxidant activity of the products against these ROS.

### Anti-inflammatory activity

**Fig 2:**
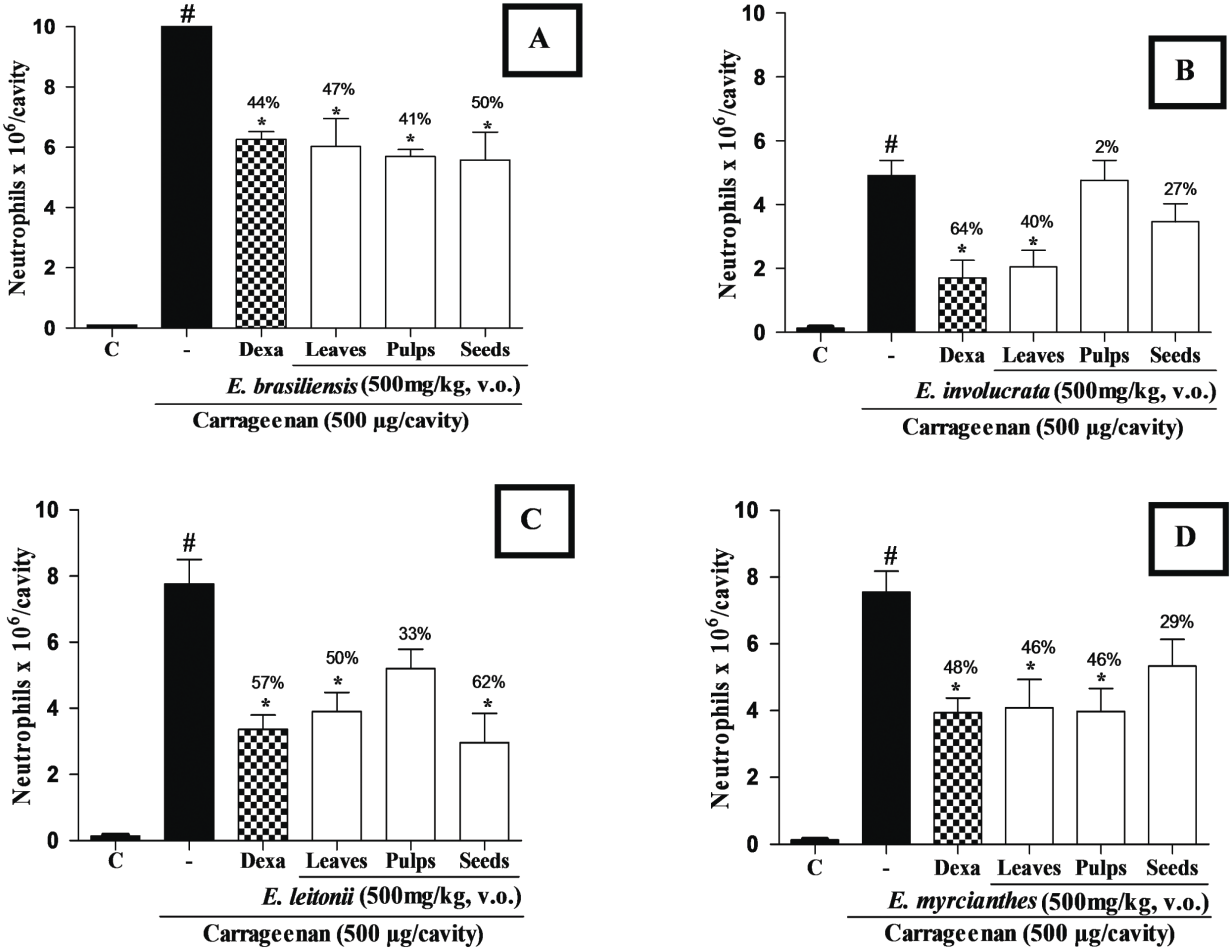
Effect of the oral administration of ethanolic extracts of leaves, seeds, and pulp of four Brazilian native fruit species on the inhibition of neutrophil migration to the peritoneal cavity of mice treated with 500 mg/kg. (A) *Eugenia brasiliensis*. (B) *Eugenia involucrata*. (C) *Eugenia leitonii*. (D) *Eugenia myrcianthes*. C: control treated with vehicle; –: carrageenan (500 μg/cavity); Dexa: dexamethasone (2 mg/kg). Data are expressed as mean ± standard deviation of the mean; n = 6. Symbols indicate statistical differences (*p* < 0.05, Tukey’s test): # *p* < 0.05 compared with the control group; * *p* < 0.05 compared with the carrageenan group. The symbol % indicates decrease in the number of neutrophils in the peritoneal cavity.

Aiming to verify the *in vivo* anti-inflammatory activity of the ethanolic extracts of leaves, seeds, and pulps of the four Brazilian native fruit species under study, the carrageenan-induced neutrophil migration assay was carried out. The results showed that the oral administration of the ethanolic extracts of leaves, pulp, and seeds of *E*. *brasiliensis* reduced the neutrophil influx by 47%, 41%, and 50%, respectively, whereas the positive control, dexamethasone, reduced it by 44% compared with the carrageenan group (Fig 2A; *p* < 0.05).

In relation to the ethanolic extract of *E*. *involucrata* leaves, the neutrophil migration was reduced by 40% and the positive control by 64% (*p* < 0.05). However, the ethanolic extracts of pulp and seeds of the same species did not significantly affect the neutrophil migration (Fig 2B; *p* > 0.05).

The ethanolic extracts of *E*. *leitonii* leaves and seeds decreased the number of neutrophils in the peritoneal cavity by 50% and 62%, respectively, and the positive control decreased it by 57% (Fig 2C; *p* < 0.05). The ethanolic extract of the pulp of the same species did not significantly affect the neutrophil migration (*p* > 0.05).

Finally, the ethanolic extracts of *E*. *myrcianthes* leaves and pulp inhibited neutrophil migration (46% for both), whereas the positive control inhibited it by 48% (Fig 2D; *p* < 0.05). The ethanolic extract of the seeds of this species did not significantly affect the neutrophil migration compared with the carrageenan group (*p* > 0.05).

The process of acute inflammation is characterized by vascular changes and leukocyte recruitment, mainly neutrophils. Additionally, the participation of inflammatory mediators released by resident cells, such as TNF-α, IL-1β, CXCL1/KC and CXCL2/MIP-2, is crucial for the development of this event [21].

During the process of inflammation, the neutrophils release hydrolytic enzymes which generate ROS, nitrogen, as well as free radicals such as hydroxyl (OH^−^) and superoxyde anion (O_2_·^−^) aiming to eliminate the aggressor. Nevertheless, in many occasions, neutrophil influx to the inflammatory site is exacerbated, leading to tissue damage; and a very high number of neutrophils constantly circulating may result in severe injury to the adjacent tissues [22–24]. Therefore, neutrophils have been found to play a key role in the pathogenesis of diseases such as atherosclerosis, obesity, rheumatoid arthritis, and periodontal disease [24].

Based on the results of the present study, we suggest that the anti-inflammatory activity of the ethanolic extracts of leaves, seeds, and pulp of four Brazilian native fruit species may be related to the modulation of neutrophil migration, as well as to the antioxidant action of the aforementioned extracts against free-radical scavenging released by the neutrophils. Phenolic compounds such as epicatechin, quercetin, and anthocyanins exhibited capacity to modulate the process of inflammation, due to their ability to decrease neutrophil migration to the inflammatory site and to block oxidative stress [25,26]. These results corroborate our study, inasmuch as the ethanolic extracts of the fruit species studied exhibited high concentrations of the phenolic compounds epicatechin and gallic acid.

## Conclusions

Our results suggest that the four Brazilian native fruit species hereby studied are excellent sources of antioxidant compounds capable of neutralizing ROS. They also exhibited good anti-inflammatory activity *in vivo*, a fact that seems to be related to the modulation of neutrophil migration and the antioxidant action of their ethanolic extracts in scavenging the free-radicals released by neutrophils. Phytochemical analyses showed that these fruit species contain high concentrations of bioactive phenolic compounds, such as epicatechin and gallic acid, suggesting that these compounds may be responsible for their antioxidant and anti-inflammatory activities. The use of fractions of these fruits, together with their leaves, is interesting for the production of new dietary supplements with health-promoting properties.

## Supporting Information

S1 Fig Appearance of the native fruit species selected for this study with botanic (and Brazilian native) names. (DOCX)

S1 Table Retention times and important ions present in the mass spectra of silylated compounds in four Brazilian native fruit species. (DOCX)
