# Supplementary material for: Antioxidant and Anti-Inflammatory Activities of Unexplored Brazilian Native Fruits
Source: PLoS One. 2016 Apr 6;11(4):e0152974. doi: 10.1371/journal.pone.0152974 (PMC4822956; doi:10.1371/journal.pone.0152974)
Supplement: S1 Fig — (DOCX) [file pone.0152974.s001.docx]

Supporting Information

*Eugenia myrcianthes Eugenia leitonii*

(ubajaí) (araçá-piranga)

*Eugenia involucrata Eugenia brasiliensis*

(cereja do rio grande) (grumixama)

**S1 Fig. Appearance of the native fruit species selected for this study with botanic (and Brazilian native) names.**
