# Supplementary material for: Antioxidant and Anti-Inflammatory Activities of Unexplored Brazilian Native Fruits
Source: PLoS One. 2016 Apr 6;11(4):e0152974. doi: 10.1371/journal.pone.0152974 (PMC4822956; doi:10.1371/journal.pone.0152974)
Supplement: S1 Table — (DOCX) [file pone.0152974.s002.docx]

Supporting Information

**S1 Table. Retention times and important ions present in the mass spectra of silylated compounds found in four Brazilian native fruit species**

| **Compounds** | | | **Retention time (min)** | **ion (m/z, abundance in parentheses)** | |
| --- | --- | --- | --- | --- | --- |
| cinnamic acid | 7,77 | | | 205(100), 131(89,6), 161(66,4), 103(56,8), 206(28); 220(M^+^) |  |
| protocatechuic acid | 9,17 | | | 370(38), 193(84,8), 355(21,6), 194(13,2), 311(10,8), 371(M^+^) |  |
| *p*-coumaric acid | 9,8 | | | 293 (100), 308(85), 219(75,2), 249(62,4), 294(26,4); 308(M^+^) |  |
| *m*-coumaric acid | 9,8 | | | 308(77,2), 293(57,2), 249(18), 139(17,2), 294(15,6); 308(M^+^) |  |
| gallic acid | 9,89 | | | 281(100), 458(93,2), 443(36,4), 282(26,8), 283(11,6); 458(M^+^) |  |
| sinapic acid | 11,7 | | | 368(100), 338(83,2), 353(49,6), 369(28,4), 323(25,2); 369 (M^+^) |  |
| 6,7-dihydroxycoumarin β-D-glucopyranoside | 14,3 | | | 361(38), 217(21,2), 169(18,8), 147(18,8), 271(11,2); 438(M^+^) |  |
| (–)-epicatechin | 17,2 | | | 368(100), 355(46), 369(34,4), 650(23,6), 370(16,4); 650(M^+^) |  |
| kaempferol | 19,34 | | | 559(100), 560(53,2), 561(24,8), 562(8), 487(7,2); 573(M^+^) |  |
| quercetin | 20,6 | | | 647(100), 648(62,4), 649(36,8), 650(11,2), 575(11,6); 662(M^+^) |  |
| myricetin | 21,64 | | | 735(100), 736(66,8), 737(41,86), 738(16,8), 647(14,4); 749(M^+^) |  |
